# Reorganisation of brain networks in frontotemporal dementia and progressive supranuclear palsy^☆^

**DOI:** 10.1016/j.nicl.2013.03.009

**Published:** 2013-03-25

**Authors:** Laura E. Hughes, Boyd C.P. Ghosh, James B. Rowe

**Affiliations:** a Department of Clinical Neurosciences, University of Cambridge, CB2 2QQ, UK; b Medical Research Council Cognition and Brain Sciences Unit, Cambridge, CB2 7EF, UK; c Wessex Neuroscience Centre, Southampton, SO16 6YD UK; d Behavioural and Clinical Neuroscience Institute, Cambridge, UK

**Keywords:** Connectivity, Dementia, Dynamic causal modelling, Magnetoencephalography, MMNm

## Abstract

The disruption of large-scale brain networks is increasingly recognised as a consequence of neurodegenerative dementias. We assessed adults with behavioural variant frontotemporal dementia and progressive supranuclear palsy using magnetoencephalography during an auditory oddball paradigm. Network connectivity among bilateral temporal, frontal and parietal sources was examined using dynamic causal modelling. We found evidence for a systematic change in effective connectivity in both diseases. Compared with healthy subjects, who had focal modulation of intrahemispheric frontal–temporal connections, the patient groups showed abnormally extensive and inefficient networks. The changes in connectivity were accompanied by impaired responses of the auditory cortex to unexpected deviant tones (MMNm), despite normal responses to standard stimuli. Together, these results suggest that neurodegeneration in two distinct clinical syndromes with overlapping profiles of prefrontal atrophy, causes a similar pattern of reorganisation of large-scale networks. We discuss this network reorganisation in the context of other focal brain disorders and the specific vulnerability of functional brain networks to neurodegenerative disease.

## Highlights

• We compare two distinct neurodegenerative syndromes with prefrontal atrophy.
• We show both diseases impair the automatic detection of change (MMNm).
• Using DCM we discover that both diseases lead to similar network reorganisation.
• We suggest principled network reorganisation in response to prefrontal degeneration.

## 1 Introduction

A key challenge to understanding the effects of neurodegeneration is to characterise the changing patterns of brain network connectivity, in response to both the disease and its treatment (Pievani et al., 2011; Seeley et al., 2009; Warren et al., 2012). The network paradigm of disease has many advantages, with the potential to elucidate selective vulnerability to a given neuropathology, explains the consequences of disease at a macroscopic level, and increases sensitivity of tools such as brain imaging that captures both integrative and segregated brain functions (Bassett and Bullmore, 2009; Mesulam, 1990). Many studies examine macroscopic networks using task-free ‘resting state’ paradigms, in which coactivation of distributed regions, or coherence among spontaneous neural oscillators, is thought to reflect functional networks (Corbetta, 2010). In response to task demands or experimental conditions, these networks are rapidly reconfigured to create a dynamic neuronal workspace for cognitive processing (Kitzbichler et al., 2011).

Neurodegenerative syndromes commonly disrupt such large scale networks (Rowe, 2010). For example, reductions in resting state connectivity mirror disease related changes in anatomical structure and connectivity with Alzheimer's disease, frontotemporal dementias and Parkinsonian syndromes (Greicius, 2008; Greicius et al., 2004; Seeley et al., 2009; Whitwell et al., 2011; Zhou et al., 2010). Task based network configuration can also be changed by focal degeneration and atrophy (Sonty et al., 2007). However, neurodegeneration not only weakens specific network connections, but can also lead to reorganisation of the networks by enhancing connectivity among the relatively unaffected regions (Seeley et al., 2008; Zhou et al., 2010). Moreover, recent studies have emphasized the concordance between reductions in network connectivity during resting state and the distinctive focal atrophy patterns in neurodegenerative dementias (Seeley et al., 2009; Zhou et al., 2010) or changes in white matter tracts supporting those networks (Raj et al., 2012).

Different clinical syndromes can result in specific changes to brain networks, but there may also be generic reorganisation in response to degeneration associated with diverse pathologies. In this study, we examined two distinct neurodegenerative diseases and asked whether disease specific neurodegeneration is related to particular network changes, or whether there is a ‘transdiagnostic’ network-level response affecting the macroscopic network dynamics.

We examined behavioural variant frontotemporal dementia (bvFTD) and progressive supranuclear palsy (PSP); two rapidly progressive neurodegenerative diseases that have key differences in clinical phenotypes. bvFTD is characterised by changes in behaviour, cognition and personality (Rascovsky et al., 2011). In contrast, PSP is defined by a vertical supranuclear gaze palsy, akinetic rigidity and falls (Litvan et al., 2003; Richardson et al., 1963), with typically milder cognitive impairment including apathy. The two syndromes have distinct and overlapping macroscopic anatomy of tissue loss. bvFTD is associated with marked atrophy of prefrontal cortex (orbital, ventral and/or medial), anterior insula, anterior cingulate and anterior temporal lobes including cortex and the amygdala (Schroeter et al., 2007, 2008; Seelaar et al., 2011; Seeley, 2010; Seeley et al., 2009), although other atrophy patterns with temporoparietal atrophy have been reported (Whitwell et al., 2009). In PSP atrophy is severe in the upper brain stem and superior cerebellar peduncle, with moderate atrophy of medial prefrontal cortex, insula/frontal operculum, cingulate cortex, precentral gyrus and superior parietal lobule (Brenneis et al., 2004; Chiu et al., 2012; Ghosh et al., 2012; Nicoletti et al., 2008).

To compare the impact of bvFTD and PSP on macroscopic *functional* brain networks, we used an auditory oddball paradigm, providing a physiological measure of automatic change detection. Such paradigms include a stream of ‘standard’ stimuli, interspersed with ‘deviant’ stimuli (e.g. differing from the standard by pitch or duration). This unpredictable change elicits a robust electrophysiological mismatch negativity signal (MMN, or MMNm in the context of MEG studies), detectable by electro- or magneto-encephalography in auditory cortex between 100 ms and 200 ms after the onset of the deviant tone. This signal has been proposed as a marker of psychiatric and degenerative conditions such as Alzheimer's disease, Parkinson's disease and schizophrenia (Naatanen et al., 2011, 2012). Moreover, from a basic science perspective, change detection is an important element of higher order cognitive functions, such as attention and memory (cf. Naatanen et al., 2007).

In addition to the auditory cortex, other brain regions contribute to the generation of the MMN response. These include prefrontal cortex (Boly et al., 2011; Doeller et al., 2003; Liasis et al., 2001; Rosburg et al., 2005; Schall et al., 2003), which is necessary for early change detection through frontal to temporal feedback connections (Alain et al., 1998; Alho et al., 1994; Garrido et al., 2009a). Parietal cortex is also associated with the MMN, in both electrophysiological (Hsiao et al., 2010; Marco-Pallares et al., 2005) and fMRI studies (Molholm et al., 2005).

To measure the network connectivity among these frontal, temporal and parietal cortical sources, we adopted dynamic causal modelling for magnetoencephalography data. Magnetoencephalography is sensitive to the spatiotemporal effects of bvFTD during cognitive tasks (Hughes et al., 2011), proportional to clinical deficits, and well tolerated by patients as a functional brain imaging modality. Dynamic causal modelling has several advantages over other methods to test our hypotheses, including (1) empirical priors that introduce biophysical constraints on the network models; (2) the use of generative (predictive) models that can be tested against the observed data, and evaluated and compared using objective measures of the model evidences; and (3) embodying different hypotheses about the impact of disease on network structures and connectivity in explicit and directional spatiotemporal network models. Dynamic causal modelling also incorporates the modulatory effects of experimental manipulations on connections, such as the difference between standard and deviant stimuli, providing evidence of the critical connections for change detection (Kiebel et al., 2006, 2007, 2008, 2009).

We used dynamic causal modelling to measure network connectivity underlying the detection of change. We included different families of network models to test two principal hypotheses. First, we predicted that the network recruited in health for change detection would be altered by disease. Specifically, since bvFTD and PSP have prefrontal neuropathology, we predicted that network reorganisation would lead to more distributed networks with enhanced connectivity among the less affected parietal regions. Secondly, we predicted that disease would also affect the modulation of the network by the experimental context (i.e. the difference between the standard and deviant tones). Thus, we not only predict that patients will have a change in network architecture, but also a change in the dynamic modulation of connectivity from trial to trial. A corollary of this network change is reduced automatic detection of unpredictable change, and therefore a reduction in amplitudes and delayed latency of the MMNm response in the auditory cortex.

## 2 Methods

### 2.1 Subjects

Seventeen patients with bvFTD were recruited using clinical diagnostic criteria, including abnormal clinical imaging, (Rascovsky et al., 2011). We did not include patients with non-progressive mimics of bvFTD (Kipps et al., 2010). Ten patients with progressive supranuclear palsy were recruited, according to clinical diagnostic criteria (Litvan et al., 1996). Subjects underwent neuropsychological assessment including: the 100 point revised Addenbrooke's cognitive examination (ACE-r) (Mioshi et al., 2006), the mini mental state examination (MMSE), the motor section of the Unified Parkinson Disease Rating Scale (UPDRS) (Fahn, 1986) and the Progressive Supranuclear Palsy Rating Scale (PSPRS, PSP cases only) (Golbe and Ohman-Strickland, 2007). Thirty-four healthy aged-matched older adults were recruited from the volunteer panel of the MRC Cognition and Brain Sciences Unit or were relatives or spouses of the patients. No subjects in the control group had a history of significant neurological, rheumatological or psychiatric illness, or cognitive complaints. Subject details are summarised in Table 1. The study was approved by the local Research Ethics Committee and participants gave written informed consent.

### 2.2 MMNm paradigm

The paradigm used to study cortical function and network connectivity was the multi-feature ‘Optimum-1’ paradigm (Naatanen et al., 2004), a variant of the auditory oddball paradigm for identification of the mismatch negativity. The stimuli comprised a sequence of harmonic tones presented every 500 ms in three blocks of 5 min. The standard tone was 75 ms duration and contained three sinusoidal partials of 500, 1000 and 1500 Hz. The five deviant tones differed from the standard by either frequency band (550, 1100, 1650 Hz), intensity (+/− 6 dB), duration (25 vs 75 ms), side of sound source (left or right rather than bilateral), and by a silent gap (silent mid 25 ms). The sequence started with fifteen standard tones, after which every other tone presented was one of the five deviant types, such that in a sequence of 10 tones, each deviant was presented once but the same deviant type was never immediately repeated. There were a total of 900 standards and 900 deviants. The tones were presented binaurally via plastic tubes and earpieces at approximately 60 dB above the hearing threshold.

### 2.3 Magnetoencephalography and data processing

MEG data were collected with a 306-channel Vectorview system (Elekta Neuromag, Helsinki) in a light Elekta-Neuromag magnetically-shielded room. A magnetometer and two orthogonal planar gradiometers were located at each of 102 positions. Vertical and horizontal eye movements were recorded using paired EOG electrodes. Head position was monitored using five Head-Position Indicator (HPI) coils. The three-dimensional locations of the HPI coils and approximately 80 ‘head points’ across the scalp, and three anatomical fiducials (the nasion and left and right pre-auricular points), were recorded using a 3D digitizer (Fastrak Polhemus Inc., Colchester, VA). Data were down sampled from 1000 to 333 Hz and pre-processed using MaxFilter software (Elekta-Neuromag) with movement compensation. The data were then processed separately for source analysis of the waveforms from auditory cortex using Brain Electrical Source Analysis (BESA version 5.3, Germany), and for network connectivity analysis using dynamic causal modelling with SPM 8.

### 2.4 Data analysis: Auditory cortical waveforms

Data were high pass filtered over 1 Hz (butterworth filter 6 dB/oct with no added padding) and the artefact rejection threshold was set to 2500 fT for magnetometers and 900 fT for gradiometers. Using BESA's adaptive artefact correction, eye-blinks were corrected and modelled in the source analysis with one fixed source. Epochs were lowpass filtered to 40 Hz (butterworth filter 24 dB/oct), time locked to the tone onset, baseline corrected (− 100 to 0 ms) and averaged from − 100 ms before tone onset to 400 ms after onset.

Source analysis of evoked responses from the gradiometer MEG channels, was performed for each subject on the MMNm response (deviant vs standard) of each deviant type, and also to the standard tone. The forward model topography (leadfield) was estimated using a realistic head model, coregistered by fiducial and digitised scalp loci. The inverse of this leadfield matrix was applied to the gradiometer data to estimate the source waveforms, varying source location and orientation iteratively until the residual difference between scalp and model waveforms was minimized. The data were fitted to two bilateral equivalent current dipoles (regional sources). The fits were constrained by imposing symmetry on the two sources, but not constrained by location or orientation, and with regularisation constant 1% to stabilise source fitting in the presence of noise.

To examine the MMNm waveforms the dipoles were fitted to an interval of 100 to 200 ms to capture the likely MMNm based on previous literature (Bronnick et al., 2010; Engeland et al., 2002; Naatanen and Escera, 2000; Naatanen and Kahkonen, 2009; Naatanen et al., 2005; Pekkonen, 2000; Pekkonen et al., 2001; Thonnessen et al., 2008). Analyses were conducted on the waveforms from the primary orientation of the regional sources. Across the 100 ms fit intervals, the average amplitude and the peak latency were estimated for the MMNm waveform of each deviant. Amplitude and latency were included as separate dependent variables in repeated measures analyses of variance (ANOVA), which had three factors: deviant type (duration, frequency, location, gap and intensity), location of the ECD (left and right hemisphere), and with group (bvFTD, PSP and controls) as a between subjects condition. Planned pairwise comparisons between the two patient groups examined differences to specific deviant types for both amplitude and peak latency (Bonferroni corrected for multiple comparisons).

To support the assumption that differences in the auditory evoked response to deviant tones would be due to impaired change detection, and not auditory perception per se, responses to the standard tone were examined. An interval of 50–150 ms was used to examine potential group differences in the M100, since differences here might reflect a hearing impairment. An analysis of variance compared the peak amplitude of the M100 for the left and right EDCs, between the three subject groups.

### 2.5 Network analysis: Dynamic causal models of cortical interactions

SPM 8 was used to prepare the data for DCM, with a similar preprocessing pipeline to the waveform analysis, except epochs in which EOG that exceeded 150 μV were rejected, to remove blink artefacts. Robust averaging was used to average all the deviant tones and separately all the standard tones. The averages of the two tone types from the gradiometer MEG channels were used for source reconstruction. A template cortical mesh was created, coregistered to the fiducial and headshape points, and used to estimate the forward model. Inverse reconstruction was computed using SPM 8's standard inversion algorithm with default settings.

We then used DCM to examine directional changes in causal influences among brain regions, in a time window of 0–250 ms. A stepwise approach to DCM was used (Stephan et al., 2010), which included 1) specifying the network by systematic variation of intrinsic anatomical connections and the connections that are modulated by experimental context; 2) fitting the data to the model by optimising model parameters; 3) estimating the free energy limit on model evidences and 4) identifying the most likely model using a hierarchical Bayesian model comparison.

The network architecture of the model space extends a previous study examining coherence among frontal, parietal and temporal sources based on the BESA auditory evoked potential model (Hughes and Rowe, 2013). Six sources were modelled using equivalent current dipoles, including: bilateral temporal nodes (MNI coordinates: +/− 43, − 21, − 4), bilateral frontal nodes (+/− 35, 33, 28), and bilateral parietal nodes (+/− 34, − 71, 13). Using these six nodes fifteen generative models were built.

The models were grouped into three families, which differed by the presence of extrinsic connections between nodes (see Fig. 2). The three families of model contained either ‘full’, ‘partial’ or ‘sparse’ bidirectional connectivity. The family of models with full connectivity had intrahemispheric connections between temporal and frontal, temporal and parietal nodes, and frontal and parietal nodes, and interhemispheric connectivity between bilateral node pairs. The family of models with partial connectivity had the same connections with the exception of the frontal–parietal connections, which were not included. The family of models with sparse connectivity had only intrahemispheric connections between temporal and frontal nodes, and temporal and parietal nodes, and no interhemispheric or frontal–parietal connectivity.

The extrinsic connections were modulated by the difference between the deviant and standard responses. Each family comprised five models, in which the connections between nodes were modulated in five ways: 1) allowing modulation to all connections, 2) to only anterior (frontal–temporal), or 3) to only posterior (temporal–parietal) connections, 4) to only forward or 5) to only backward connections. For all models, the driving cortical input was specified at left and right auditory cortex. Intrinsic connectivity within each of the temporal nodes was also modulated, in accordance with a prior DCM study of a roving oddball paradigm (Garrido et al., 2008, 2009b).

To establish the best model for each group of subjects, we used Bayesian model comparison cf. (Fastenrath et al., 2009; Kiebel et al., 2009; Stephan et al., 2009) for random-effects inferences that accommodate heterogeneity within group. The free energy estimates of the bound on model evidences (adjusted for model complexity and covariance amongst parameters) were used to estimate the model exceedance probability (the probability that a model or family of models was more likely than any other model or family, to have generated the data) and the expected posterior model probability (the probability of that model, or family of models, given the observed data).

This model comparison was completed in a two stage process. First, we identified the most likely anatomical network involved in responding to standard and deviant tones, by comparing the ‘full’, ‘partial’ and ‘sparse’ families of models cf. (Penny et al., 2010). Secondly, we examined how the most likely network was modulated in response to the deviant vs standard tones. The five models that comprised the most likely family were compared to provide a single model for each group which describes how the connections were modulated. We report the model comparisons using the exceedance probability and expected posterior probability. Note that the Bayesian inference implicit in model selection makes a positive statement about the probabilities of models (within the set of 15 models tested) given the data, and do not make frequentist statistical inferences about the (un)likelihood of the data given a null hypotheses (Kass and Raftery, 1995; Raftery, 1995), obviating multiple comparison corrections.

## 3 Results

### 3.1 Auditory cortical waveforms: The M100

**Fig. 1:**
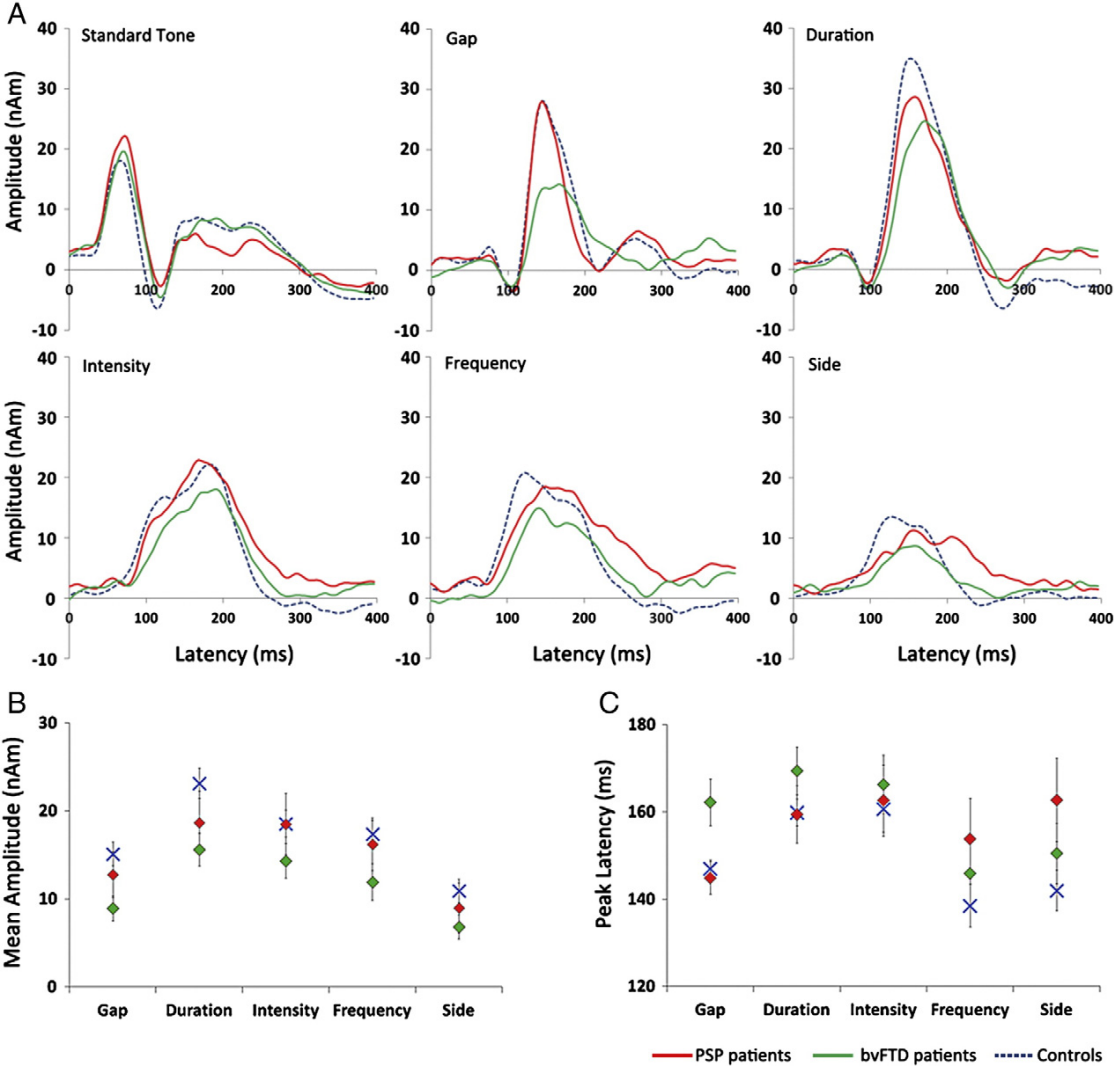
A) Waveforms from the two-dipole model, for the standard tone (75 ms, combining 500 Hz, 1000 Hz and 1500 Hz sinusoids) and the mismatch negativity for each of the five deviant tones for bvFTD and PSP patients and controls. B) Mean amplitude across 100–200 ms of the MMN waveforms. Reduced mean amplitude of response is clear across all deviants for bvFTD and most deviants for PSP patients, compared to controls. C) Peak latency of MMN waveforms. Latencies are delayed for the patient groups, and specifically for the gap deviants in bvFTD patients.

The maximum peak amplitudes, representing the M100 for the standard tone, within the 50–150 ms window were compared between the bvFTD, PSP and control groups. There were no significant differences between the three groups for left and right sources, suggesting normal early auditory processing of the standard tone (F_(2,58)_ = 1.2, *p* > 0.05). See Fig. 1A and Table 2.

### 3.2 Auditory cortical waveforms: Mismatch negativity

For each deviant type, the average MMNm amplitude across the 100–200 ms window and the latency of the peak amplitude were calculated (Fig. 1A). Two separate analyses of variance were used to investigate differences in mean amplitude and peak latency between bvFTD, PSP patients and controls. Each ANOVA had three factors: deviant type (frequency, intensity, duration, side and gap), laterality of dipole (left and right), and subject group (bvFTD, PSP and controls).

There were significant differences in mean amplitude between the three subject groups, with the patient groups having reduced amplitudes (F_(2,58)_ = 5.1, *p* = 0.009), but there were no significant interactions with deviant type, nor with dipole location (All F < 1). The pairwise comparisons between the bvFTD and PSP patient groups revealed no significant differences. See Fig. 1B.

The ANOVA of peak latencies also revealed significant differences between the three groups, with the patient groups having later peaks than the controls (F_(2,57)_ = 3.4, *p* = 0.037) but there were no interactions with deviant type, nor with dipole location (All F < 1.7). The pairwise comparisons between the patient groups showed that the latencies for the bvFTD patients were significantly delayed compared to the PSP patients for the gap deviant (t_(25)_ = 3, *p* = 0.005 Bonferroni corrected). See Fig. 1C.

### 3.3 Network connectivity analysis (dynamic causal modelling)

**Fig. 2:**
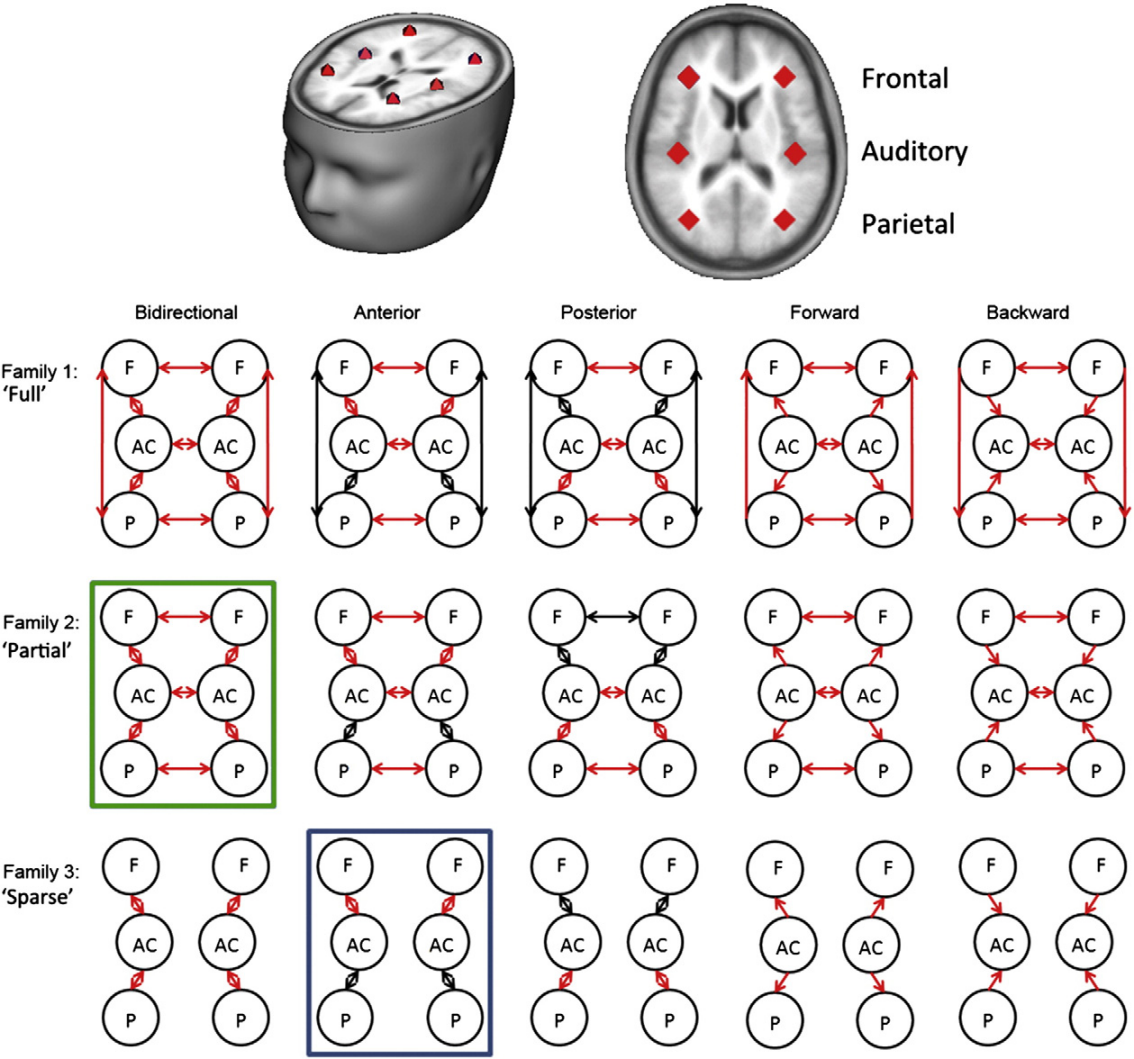
Model specification for DCM. Fifteen network models were compared. Each had six nodes fitted to bilateral frontal (F), auditory (AC) and parietal (P) cortical sources. The models are differentiated in three ways by the connections between the nodes: First, by the intrinsic connections: These connections can be between all nodes (as in Family 1), between a partial set of nodes (as in family 2) or the connections are sparse (as in Family 3). Second, the models can be differentiated by the direction of the connections (bidirectional, forward or backward connections, as indicated by the arrows). Third, the strength of the connection can be modulated by the difference between the standard and deviant tones. These modulated connections are depicted in red and either all connections are modulated, or just anterior or posterior connections, or just forward or backward connections. The model with a green surround is the most likely for both groups of patients, and with a blue surround is the most likely for controls.

**Fig. 3:**
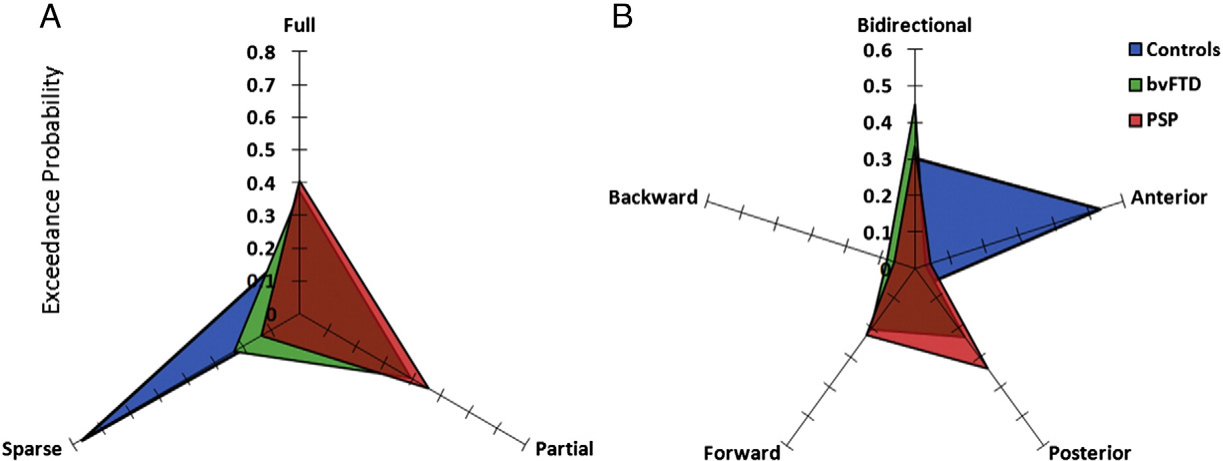
A) Model exceedance probability for comparison of fifteen models, grouped by family of extrinsic connections (full connectivity between nodes, partial connectivity, sparse connectivity). For controls (blue) the Sparse models are more likely, whereas for the two patient groups the models with partial connections are more likely. B) Exceedance probability for the five models within the best family for each group of subjects. For controls the sparse model in which the anterior forward and backward connections are modulated by the deviant tone different is most likely, but for both groups of patients, the model of partial connections, in which all bidirectional connections are modulated is most likely. Notably, the worst model for the patients is the model in which just anterior connections are modulated.

In the first stage of model comparison, 15 models were grouped into three families of five models. The three families had either ‘full’, ‘partial’ or ‘sparse’ extrinsic connectivity (Fig. 2). For the control group, the most likely model family was the sparse network, (exceedance probability, *p* = 0.77, posterior probability, *p* = 0.46) in which there were bidirectional intrahemispheric connections between temporal and parietal nodes and between temporal and frontal nodes, but no interhemispheric connections, or frontal–parietal connectivity. For the patients with bvFTD and PSP, the model family with sparse connectivity was least likely (exceedance probability, *p* = 0.24 and *p* = 0.13, posterior probability, *p* = 0.3 and *p* = 0.24 respectively). The models with a partial network connectivity, defined by interhemispheric connections in addition to the frontal–temporal and temporal–parietal connections, were most likely for both patient groups. The partial models for the bvFTD and PSP patient groups had an exceedance probability of *p* = 0.38 and *p* = 0.45, posterior probability, *p* = 0.35 and *p* = 0.38 respectively (see Fig. 3A for contrasting model family probability for each group).

The second stage of our hierarchical model comparison examined how connectivity was modulated by the difference between the standard and deviant tones, and included the 5 models within the most favoured family for each group of subjects. For the controls, within the family of sparse models, the model with anterior bidirectional connections was favoured (exceedance probability, *p* = 0.53, posterior probability, *p* = 0.29). For patients with bvFTD, within the family of partial connectivity, the model in which all bidirectional connections were modulated was the favoured model (exceedance probability, *p* = 0.44, posterior probability, *p* = 0.29). For patients with PSP two models from the family with partial connectivity fit the data equally: the model in which all bidirectional connections were modulated and the model in which just posterior bidirectional connections were modulated (exceedance probability, *p* = 0.33, posterior probability, *p* = 0.27). See Fig. 3B for contrasting individual model probabilities for each group. Notably however, for the two patient groups, the models with just anterior frontal–temporal modulation were the worst fit for the data (exceedance probability, *p* = 0.03 and *p* = 0.04, posterior probability *p* = 0.11 and *p* = 0.11, respectively), suggesting that in contrast to the control group data, a sparse network of frontal–temporal connectivity is unable to reliably account for the observed data.

### 3.4 Correlations with behavioural analyses

Multiple regression models were used to examine whether the changes observed in the evoked response and connectivity with disease were related to measures of disease severity. In the first model for each of the patient groups the global ACE-r score was entered as the dependent variable and the predictor variables included the mean amplitude of the MMNm (collapsed across deviant types) and the frontal, temporal and parietal interhemispheric connectivity measures from the most likely ‘partial’ network model. These measures were selected since the interhemispheric connections were the defining difference between the most likely family models for the patient and control groups. In the bvFTD group these MEG and DCM metrics did not significantly predict ACE-r scores. In PSP temporal interhemispheric connectivity significantly predicted the ACE-r score (β = − .67, *t*(5) = − 2.7, *p* = .04), suggesting that higher temporal interhemispheric connectivity is associated with poorer ACE-r in PSP. The model explained a significant proportion of the variance of scores (*R*^2(adj)^ = 0.6, F_(5,9)_ = 5, *p* = 0.05). Previous studies of different disease types have indicated that neuropsychological scores associated with frontal lobe executive functions, (such as verbal fluency), are related to frontal electrophysiological components, (Higuchi et al., 2013; Soininen et al., 1995; Thomas et al., 2010) and verbal fluency is particularly sensitive to differentiating Parkinsonian syndromes (Rittman et al., 2013). Therefore a new regression model was used to test whether verbal fluency was associated with the MEG and DCM metrics. For the bvFTD group, all predictor variables were significant: (amplitude: *β* = .26, *t*(11) = 2.6, *p* = .026; frontal interhemispheric connectivity: *β* = 17.5, *t*(11) = 2.6, *p* = .02; temporal interhemispheric connectivity: *β* = − 9.5, *t*(11) = − 2.2, *p* = .046; parietal interhemispheric connectivity *β* = 10.9, *t*(11) = 2.6, *p* = .025), suggesting that increased MMNm amplitude, frontal and parietal interhemispheric connectivity, and reduced temporal interhemispheric were associated with better fluency. The model also explained a significant proportion of the variance of fluency scores (*R*^2(adj)^ = 0.47, F_(4,15)_ = 4.4, *p* = 0.02). In the PSP group these MEG and DCM metrics did not significantly predict verbal fluency.

### 3.5 Pharmacological effects

To address a potential confound of serotonergic medication in bvFTD, post-hoc analyses were conducted comparing bvFTD patients taking serotonergic medications (n = 6, SSRI +) with those who were not (n = 11, SSRI −) (see Table 1). The ANOVAs of the waveforms were repeated, and collapsed across laterality of dipole, with the serotonin bvFTD sub-groups as a between subject factor. No significant differences between the groups were revealed for either amplitude (F_(1,15)_ = 0.9, ns) or peak latency (F_(1,15)_ = 1.0, ns) measures. There were no significant interactions between deviant type and subject group for amplitude (F_(4,60)_ = 2.0, ns) or latency (F_(1,15)_ = 0.8, *p* > 0.05).

The Bayesian model comparison was repeated for the SSRI + and SSRI − groups, and showed that the most likely model within the winning ‘partial’ network family was unchanged for both subgroups; i.e. the model in which all bidirectional connections were modulated by trial type.

## 4 Discussion

This study confirms our principal hypothesis, regarding changes in functional brain networks caused by frontotemporal dementia and progressive supranuclear palsy. There was a common pattern of reorganisation in response to both neurodegenerative diseases, with the recruitment of a more extended network in response to auditory stimuli. There were additional abnormal interactions among frontal, temporal and parietal cortex, which were all modulated by the contextual difference between the standard and deviant tones. Despite this extended network there was a concomitant reduction in the temporal cortical responses to unpredictable stimuli: both amplitude and latency of the MMNm response to deviant stimuli were affected.

In healthy older adults, there was evidence in favour of a sparse network with focal anatomical connectivity and restricted contextual modulation of only anterior frontal–temporal connections. This concurs with previous studies using DCM of mismatch negativity. For example, in young adults with a roving oddball paradigm, Garrido et al. (2008, 2009b) found that the most likely network had bidirectional frontal–temporal connections, with connectivity modulated by the difference between the standard and deviant tones. Despite minor differences in task and network models, the organisational principles of the likely brain network were similar across studies: sparse and efficient connectivity for change detection.

In contrast, both patient groups were associated with a very different network model as the most likely, given the observed data. The focal intrahemispheric networks were supplemented by interhemispheric connections, together with contextual modulation of all connections, including temporal–parietal connections, in response to deviant stimuli. The comparison of model evidences did not distinguish between bvFTD and PSP, at either the family level of network architecture, or at the level of modulation of connections in response to change detection. This suggests that disruption within the network can result in a generic shift of connectivity, leading to a similar response in terms of an inefficient and distributed network in both diseases.

In the two patient groups the strength of interhemispheric connections was related to measures of disease severity; more specifically increased temporal connectivity predicted poorer neuropsychological performance in both groups (ACE-r in PSP and verbal fluency in bvFTD) suggesting that this deviation from the pattern in health is a function of the disease process. In bvFTD, increased frontal and parietal interhemispheric connectivity also predicted better verbal fluency, which may potentially be compensatory. Previous research also indicates that neuropsychological performance correlates with changes in electrophysiology: including relationships between frontal neuropsychological tests and P50 gating (Thomas et al., 2010); habituation of the N100 and verbal fluency (Soininen et al., 1995) and MMN amplitude and memory (Ruzzoli et al., 2012).

The similar patterns of network connectivity in bvFTD and PSP might be explained by the partially overlapping profiles of prefrontal cortical atrophy and dysfunction in both diseases, despite diverse patterns of underlying neuropathology (Josephs et al., 2006; Mackenzie et al., 2011; Rohrer et al., 2011; Seelaar et al., 2011), although the atrophy is less severe in the frontal lobes of PSP than bvFTD. The network similarity may also reflect the overlap of cognitive and behavioural impairments (Ghosh et al., 2012; Yatabe et al., 2011). Both diseases are associated with executive dysfunction (Bak and Hodges, 1998; Grafman et al., 1995; Millar et al., 2006), and poor social and emotion cognition (Ghosh et al., 2009, 2012; Keane et al., 2002; Rankin et al., 2005; Schrag et al., 2003). However, the two disorders are phenotypically distinct, at least in the study populations included here, despite reports of overlapping syndromes, occasional intermediate phenotypes or diagnostic evolution (Kertesz et al., 1999, 2011).

The change in connectivity, from the sparse network model observed in health to a more distributed network in the patient groups, has two aspects of particular interest. The first is the inclusion and modulation of additional temporal–parietal connections. This accords with observations from resting state studies which also demonstrate a posterior shift in activity with degenerative diseases that affect frontal cortex (Hughes and Rowe, 2013; Seeley et al., 2008; Zhou et al., 2010). This shift of increased connectivity in intact networks following reduced connectivity of damaged networks is reminiscent of the widespread changes observed after other forms of brain insult. Such distributed reorganisation of macroscopic network connectivity, even remote from the regions of direct injury, has been associated with many different neurological conditions including stroke (Sharma et al., 2009), tumour excisions (Rowe et al., 2007) and Parkinson's disease (Rowe et al., 2010).

The mechanism of this network reorganisation remains to be established, although our data are not sufficient to determine this. The posterior shift may be compensatory for loss of function in anterior regions, considering the relative integrity of posterior parietal lobes in both diseases despite any underlying pathological differences. Alternatively, the shift may reflect relative disinhibition of posterior networks following frontal lobe dysfunction and loss of top down control. In patients who have focal lesions after stroke, rather than focal degeneration, recruitment of additional regions can be indicative of poorer recovery (Ward et al., 2003a, 2003b). Thus increased activity in abnormal regions during degenerative disease may be a marker of decline. Whether the extension of connected networks in response to frontal or frontotemporal degeneration is compensatory or not, it clearly distinguishes the patient populations from controls: the most likely network model for the control group was the least likely for the patient groups.

The second feature of interest in the network change is that it is observed in the early stages of auditory processing. The MMNm component is an early (between 100 and 200 ms), automatic or largely pre-attentive response (Naatanen et al., 2007). Particularly relevant is that a prefrontal contribution to change detection also occurs within this early time window, demonstrated in Garrido and colleagues' dynamic causal modelling of a roving oddball paradigm (Garrido et al., 2008) and the effects of frontal lesions (Alho et al., 1994). This suggests that if the prefrontal cortical dysfunction in the two patient groups is the cause of the altered network response, then this affects the very early stages of stimuli processing. Although this early component is distinct from the later M300 response, which reflects attentional processing (Wronka et al., 2008), it is still relevant to high order cognitive processes, (Naatanen et al., 2007, 2012).

Despite the similarities in the changes of network connectivity, differences between the groups were revealed in the evoked responses measured at the auditory cortex. The changes in waveform analyses of the mismatch negativity cannot be attributed to simple hearing impairments: The patient groups did not differ in their M100 response to the standard tone.

For the two patient groups, the mean amplitude of the MMNm was reduced and the peak latencies were delayed compared to the control group suggesting a global dysfunction of change detection. Comparisons of the two patient groups revealed the bvFTD patients had delayed peak responses especially to the gap deviant. This deviant type relates to integrating information across time since the gap deviant has a brief silent pause after 25 ms, compared to the continuous standard tone of 75 ms. Poor time estimation, or a slow internal clock, is described in neurodegenerative disease such as Parkinson's disease (Pastor et al., 1992), and in patients with focal prefrontal lesions (Casini and Ivry, 1999), and has been related anatomically to the prefrontal cortex cf. (Coull et al., 2011) which may explain the delayed response in bvFTD to this specific deviant type, considering the greater extent of frontal atrophy in these patients.

There are a number of limitations to the study. We have grouped our patients into bvFTD and PSP based on clinical diagnostic criteria, however it is likely that the underlying pathologies may differ between individual patients within each group. For PSP, clinicopathological correlations are high, with characteristic tau pathology in excess of 90% of cases. In bvFTD, the pathological correlations are poorer, and sporadic cases may be due to tau or ubiquitin pathology in approximately equal measure (Whitwell et al., 2012). Despite the likely pathological heterogeneity in our group of bvFTD patients, they form a coherent clinical syndrome based on the neurocognitive systems that are most damaged by degeneration. Other factors may also contribute to the differences we observe, for example the patients in each group are in different stages of disease progression.

Medication is also a potential confound. A number of pharmacological studies have examined the role of different neurotransmitters on the MMN, demonstrating both reductions and enhancements in amplitudes for serotonin and to a lesser extent dopaminergic medications (Kenemans and Kahkonen, 2011) (although see (Leung et al., 2010)). In bvFTD particularly these are important considerations, since there are marked reductions in serotonergic innervation of prefrontal cortex (Huey et al., 2006; Salmon, 2007; Yang and Schmitt, 2001). Although the post-hoc analysis of bvFTD patients taking serotonin reuptake inhibitors/agonists was not indicative of any significant effects, the current data set is not sufficient to resolve considerations of pharmacological effects on the mismatch negativity (Leung et al., 2008; Wienberg et al., 2010), which would require a larger and placebo controlled design.

In considering the results of dynamic causal modelling, we stress that we evaluated a systematically constructed set of models which embodied our hypotheses, but we did not test all possible networks: this is both unnecessary and intractable computationally (Stephan et al., 2010). The model set was sufficient for our principal hypotheses and allowed us to examine cortico-cortical network changes related to the change detection paradigm. However, it should be noted that the biophysical model of dynamic causal modelling includes assumptions about the distinctions between feedforward, feedback and lateral connections, in terms of laminar architecture of cortex. There is a degree of laminar specificity to FTD and PSP pathology (Armstrong and Cairns, 2009; Armstrong et al., 2012), but we do not over extend our modelling or inferences to make claims about the specific intracortical aetiology of the connectivity changes we identify. Finally, we note that our analyses do not in themselves show progression of network connectivity changes with time, or in response to treatment. These important issues call for further work, before such network modelling or magnetoencephalography can be used as diagnostic, prognostic or therapeutic biomarkers in bvFTD or PSP, although parallel studies in other degenerative diseases are promising in this direction (Zamrini et al., 2011).

## 5 Conclusion

We have shown that two neurodegenerative syndromes, bvFTD and PSP, cause a similar change in the organisation of large-scale functional brain networks. The sparse intrahemispheric network with modulation of frontal–temporal connectivity was replaced by a widely distributed network with interhemispheric connectivity, and modulation of frontal–parietal and temporal–parietal connections. These network changes were accompanied by reduced amplitudes and delayed latencies of the MMNm. The similarity of the response of the neural networks to two clinically distinct disorders may reflect commonalities in their neuropathology, and is likely to be a response to focal degeneration in prefrontal cortex. This response includes recruitment to the network and more extensive, albeit inefficient, contextual modulation of connectivity. Given the importance of change detection to many higher cognitive functions, including learning and goal-directed behaviours, dysregulation of its underlying network may contribute to the higher order cognitive deficits observed in bvFTD and PSP. However, before these methods can be adopted as biomarkers for dementias, further work will be required to show the temporal evolution of plexopathy, and the extent to which it can be reversed by candidate therapies.

## Figures and Tables

**Table 1:** Mean summary scores for the patient groups and controls.

	Age	F/M	Age of onset	Years diagnosis	MMSE^a^	ACER^b^	ACER subscales					CBI^d^	PSP rating	UPDRS^e^	Medication	
						Total	Attention	Memory	Fluency	Language	VSp^c^				SA^f^	DA^g^
bvFTD	60.2	8/9	56.1	3.8	23.2	67.8	14.5	15.4	3.6	21.6	12.8	104	~	~	6	3
n = 17	(6.9)		(8.2)	(2.1)	(4.2)	(14.1)	(2.9)	(6.8)	(2.9)	(4.1)	(2.9)	(50)				
PSP	67.5	2/8	64	4.2	26.5	75.8	16.2	19.6	4.4	22.6	11.7	69.5	27.8	26.6	3	7
n = 10	(7.3)		(8.1)	(1.5)	(3.2)	(17.6)	(1.4)	(6.9)	(3.2)	(5.7)	(5)	(28.8)	(9.7)	(12.5)		
Controls	65.7	15/19														
n = 34	(7.4)															

a MMSE: 30-point mini mental state examination.

b ACER: 100 point Addenbrooke's cognitive examination revised.

c VSp visuospatial subscale.

d CBI: Cambridge Behavioural Inventory (Wedderburn et al., 2008).

e UPDRS: Unified Parkinson's Disease Rating Scale.

f SA: Serotonin agonists/reuptake inhibitors, including citalopram, venlafaxine, and trazodone.

g DA: Dopamine agonists/reuptake inhibitors, including Madopar, Sinemet and Amantadine. Standard deviation in parenthesis.

**Table 2:** Mean peak amplitudes (nAm) representing the M100 from left and right dipoles fitted across a 50–150 ms window for the three subject groups. Standard errors are in parenthesis.

	Left dipole	Right dipole
Controls	− 10.4 (1.9)	− 12.1 (1.8)
bvFTD	− 10.6 (2.7)	− 11.6 (2.6)
PSP	− 4.1 (3.5)	− 7.2 (3.4)
